# Case Report: Intravesical and extravesical urachal cyst in children with lower abdominal pain and hematuria

**DOI:** 10.3389/fped.2024.1410976

**Published:** 2024-06-03

**Authors:** Kaiyi Mao, Leibo Wang, Yuchen Mao, Xianhui Shang, Guangxu Zhou, Peng Zhao

**Affiliations:** ^1^ Department of Pediatric Surgery, Affiliated Hospital of Zunyi Medical University, Zunyi, China; ^2^ Department of Pediatric Surgery, Guizhou Children's Hospital, Zunyi, China; ^3^ Urology Surgery, Beijing Jishuitan Hospital Guizhou Hospital, Guiyang, China

**Keywords:** urachal cyst, intravesical urachal cyst, children, hematuria, case report

## Abstract

Bladder urachal cysts in children are a rare form of urachal abnormality. In this paper, we present a case of atypical imaging that presented with lower abdominal pain accompanied by hematuria, resulting in the formation of both internal and external urachal cysts in a child. A 6-year-old male child presented with repeated abdominal pain over a span of 4 days. Color ultrasound and pelvic CT scans revealed a soft tissue lesion on the right anterior wall of the bladder with an unclear boundary from the bladder wall. Voiding Cystourethrography (VCUG) showed no significant abnormalities in the bladder, while routine urine testing was positive for hematuria. A cystoscopy was simultaneously performed with a laparoscopic resection of the urachal cyst. Intraoperative cystoscopy identified the intravesical lesion, which was precisely removed using a cystoscope-assisted laparoscopy. Postoperative pathology confirmed that both extravesical and intravesical lesions were consistent with a urachal cyst. No complications were observed after the operation, and no recurrence was noted during a six-month follow-up. Therefore, for urachal cysts at the bladder's end, the possibility of intravesical urachal cysts should not be excluded, especially in patients with microscopic hematuria. We recommend performing cystoscopy simultaneously with laparoscopic urachal cyst removal to avoid missing intravesical lesions.

## Introduction

The urachus, also known as the median umbilical ligament, is a tubular structure that connects the umbilical cord to the front wall of the bladder (1). During the fourth to fifth month of pregnancy, the bladder gradually descends into the pelvis, and the urachal lumen closes and disappears, thus forming a permanent fibromuscular cord (2). However, abnormalities in embryonic development can lead to urachal anomalies (UA), which are rare pediatric urinary diseases, particularly for males (3). Congenital UA can be divided into five main types of persistent urachal remnants according to the patency of the duct:patent urachus, urachal sinus, urachal diverticulum, urachal cyst, and alternate sinus. (1) A patent urachus is an open sinus connecting the bladder to the umbilicus. (2) Urachal cyst is a cyst along the urachal duct, which is mainly located in the middle of the duct, which has obliterated cranially and caudally. (3) Urachal sinus may communicate with the umbilicus but not the bladder or with the bladder but not the umbilicus. (4) The development of a urachal diverticulum occurs when a vesical end of the urachus fails to obliterate and the umbilical end experiences complete obliteration. (5) The alternating sinus is a cystic dilatation of the urachus that periodically empties into the bladder or the umbilicus. Urachal cyst (UC) comprising 31%–43% of cases (4, 5). Usually, urachal cysts are located outside the bladder. However, intravesical urachal cysts are a rare type of UA (6).

## Case presentation

**Figure 1:**
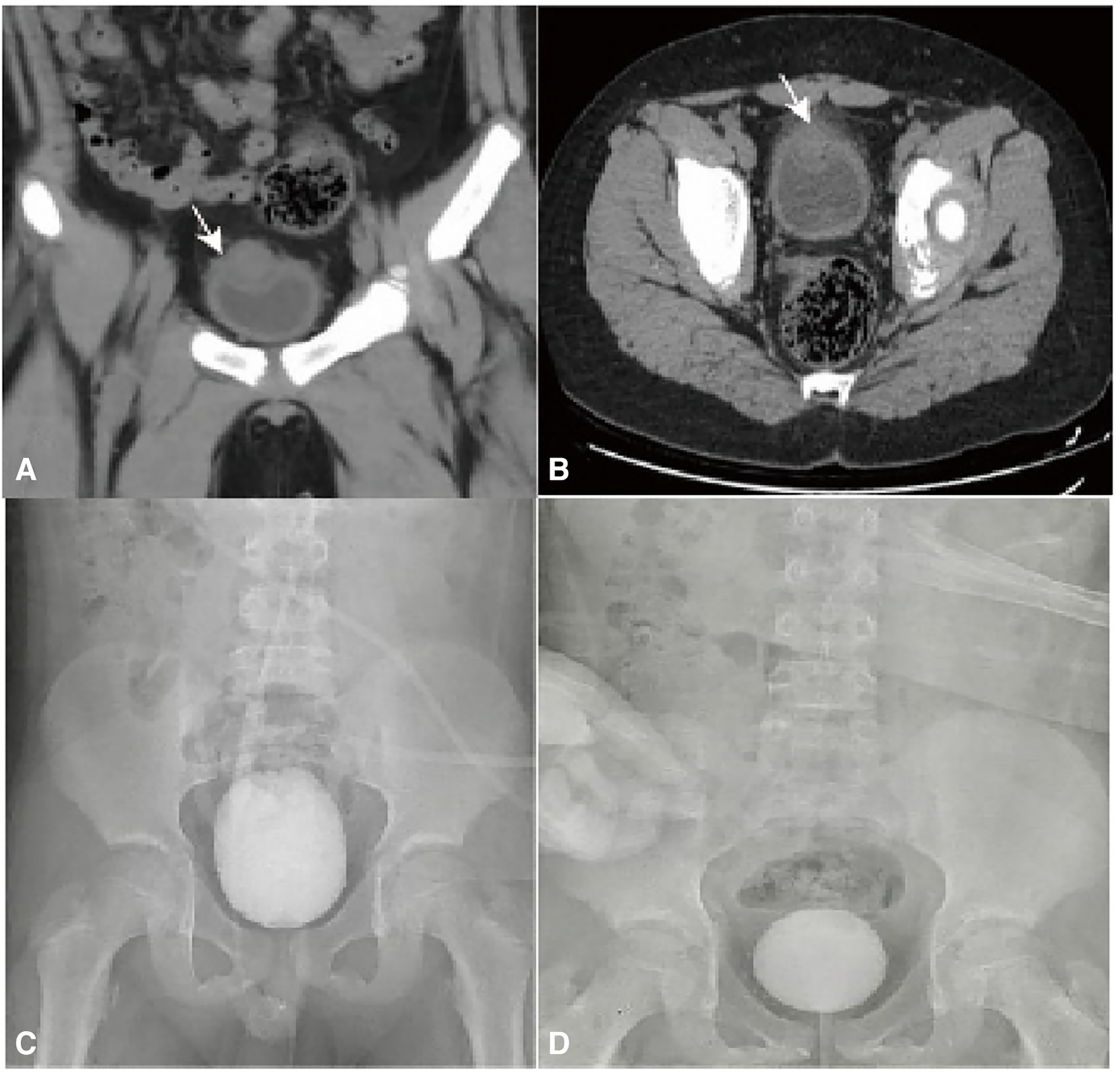
(**A,B**) CT scan revealed a mass of soft tissue density in the urachal area at the anterior upper edge of the bladder, approximately 32 mm × 33 mm × 29 mm in size, with unclear boundary with the bladder (arrow). (**C,D**) VCUG findings: No filling defects or diverticula were observed during bladder filling and emptying, and there was no reflux in the bilateral ureters.

**Figure 2:**
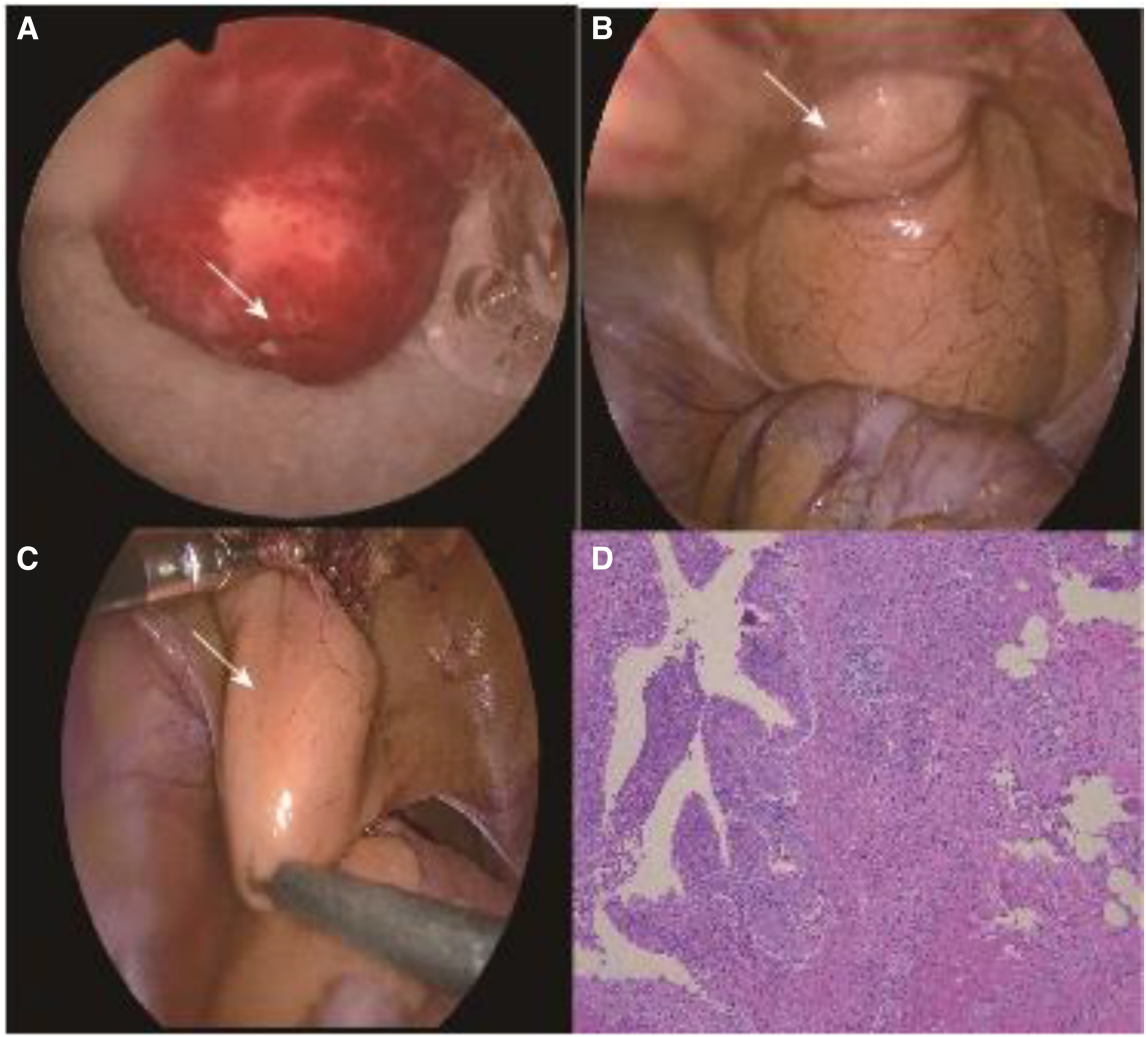
(**A**) Intraoperative cystoscopy revealed a round lesion near the bladder neck, measuring approximately 8 mm × 6 mm, with a central fistula (arrow). (**B**) A mass was observed at the top of the bladder during laparoscopy, measuring approximately 28 mm × 25 mm (arrow). (**C**) Cystoscope-assisted laparoscopic provides surgical area positioning for surgery (arrow). (**D**) Postoperative pathology showed that the lesions in the bladder and those outside the bladder were consistent with a urachal cyst with acute and chronic suppurative inflammatory changes.

A 6-year-old male patient was admitted to the hospital with recurrent abdominal pain for 4 days, mainly in the lower abdomen, presenting as dull pain. Upon physical examination, lower abdominal tenderness was noted, with no rebound pain, no palpable mass, and no redness, swelling, or secretion from the umbilicus. Additionally, no obvious abnormalities were found during the physical examination. The urine routine showed 17 white blood cells/µl and 45 red blood cells/µl. Color ultrasound of the urinary system revealed an isoechoic mass in the right anterior wall of the bladder with a regular shape, an unclear boundary with the bladder wall, and no obvious blood flow signal within it, suggesting the possibility of a urachal cyst. Pelvic CT revealed a soft tissue mass in the urachus area at the anterior upper margin of the bladder wall, measuring approximately 32 mm × 33 mm × 29 mm in maximum cross-section. The boundary between the lower margin and the bladder was unclear, prompting consideration of infectious or space-occupying lesions (Figures 1A,B). VCUG revealed no abnormality in bladder shape or size, no filling defect or niche in the bladder, and no bilateral ureteral reflux (Figures 1C,D). Based on the symptoms and related imaging data of the child, the initial preoperative consideration was that the urachal cyst outside the bladder wall was complicated by infection. Considering the child's microscopic hematuria, a decision was made by the treatment team to perform cystoscopy in addition to laparoscopic urachal cyst resection to determine the presence of bladder lesions. During the operation, cystoscopy revealed a circular mass near the top of the bladder neck, measuring 0.8 cm*0.6 cm, with a fistula in the middle and a small amount of blood clot attached to the surface (Figure 2A). Exploration with an F3 ureteral catheter encountered resistance after entering approximately 0.8 cm. Subsequently, laparoscopy revealed a hard mass at the top of the bladder (Figure 2B), measuring about 28 mm × 25 mm. The lesion was completely resected down to the bladder detrusor muscle layer without entering the bladder mucosa layer. After a second cystoscopy, the bladder lesions were still present. As a result, foreign body forceps were used to clamp the tumor and gently push it outward to facilitate localization of the operative area for laparoscopy (Figure 2C). Following cauterization and positioning with an electric coagulation hook, part of the bladder tissue was removed together with the bladder urachal cyst. The wound was sutured continuously with 4–0 absorbable suture to close the bladder tear, followed by discontinuous embedding of the seromuscular layer. Postoperative pathology showed that the lesions in the bladder and those outside the bladder were consistent with a urachal cyst with acute and chronic suppurative inflammatory changes (Figure 2D). The postoperative course indicated that the patient recovered well, with the catheter removed on the 5th day after surgery. The patient was discharged 7 days after surgery, without complications such as intestinal obstruction. During a follow-up period of half a year, the patient exhibited normal urination, no abdominal pain, hematuria, and no recurrence of bladder diverticula found by color ultrasound.

## Discussion

Generally, most urachal cysts are not connected to the bladder or umbilicus; they are exogenous to the bladder. However, urachal cysts inside the bladder protrude into the bladder, making this type of cyst very rare (1, 6). In 2013, Metwalli et al. (6) first proposed the concept of “intravesical urachal cyst” and described its color ultrasound manifestations: An intravesical urachal cyst presents as a thin-walled oval cystic structure protruding into the lumen above the anterior midline of the bladder wall. The researchers observed a cystic filling defect in the bladder dome area after VCUG in a child. However, in this case, the results of the color ultrasound did not match the typical manifestations of the urachal cyst in the bladder. There were also no abnormalities observed in VCUG, which may be caused by the fact that the urachal cyst had less tissue protruding into the bladder, making it easy to be ignored during imaging examinations.

In this case, the child's urine routine indicated microscopic hematuria, which led us to highly suspect the presence of lesions in the bladder. During intraoperative cystoscopy, our suspicions were confirmed. A 0.8 cm*0.6 cm round mass was found near the top of the bladder, close to the bladder neck, exhibiting a fistula in the middle with a small amount of blood clot attached to the surface, which was the cause of the hematuria. Z Alyusuf et al. (7) also reported a case of a bladder urachal cyst suspected of rhabdomyosarcoma. The main symptoms in that child were hematuria and urinary tract infection. The color ultrasound examination revealed clear boundary soft tissue lesions at the top of the bladder with internal blood vessels. Ultimately, the postoperative pathological diagnosis confirmed a urachal cyst. In contrast to the present case, the results of the color ultrasound showed a well-defined echogenic mass with a regular shape on the right anterior wall of the bladder, indistinguishable from the bladder wall, with no obvious blood flow signal detected. Without cystoscopy during the operation, it would not be easy to overlook the urachal cyst in the bladder. This could result in the lesion not being completely removed, leading to no significant improvement, or even the exacerbation of symptoms, or potentially progressing to cancer in children after surgery. The treatment of congenital urachal abnormalities remains controversial. While the risk of malignant transformation is low, the traditional approach involves surgical resection (8). Some scholars advocate for conservative methods in asymptomatic or mild cases, suggesting that urachal cysts may spontaneously resolve (9). On the other hand, other scholars argue that (10) surgical removal is clearly indicated for large or suspicious urachal lesions, regardless of symptoms. Laparoscopic surgery is a safe and feasible method, offering advantages such as a shorter hospital stay, quicker recovery, and less bleeding (11).

In conclusion, for urachal cysts at the bladder end, especially in patients with microscopic hematuria, the possibility of intravesical urachal cysts cannot be ruled out. Even if the imaging examination indicates no abnormalities in the bladder, it is recommended to perform cystoscopy simultaneously with laparoscopic removal of urachal cysts to rule out the possibility of intravesical lesions. If the lesion is found in the bladder, it can be accurately located with the assistance of a cystoscope, and the cyst in the bladder can be accurately removed by laparoscopy to avoid the psychological burden and economic pressure brought by missed diagnosis and second surgery.

## Data Availability

The original contributions presented in the study are included in the article/Supplementary Material, further inquiries can be directed to the corresponding author.
